# Mutations in calmodulin-binding domains of TRPV4/6 channels confer invasive properties to colon adenocarcinoma cells

**DOI:** 10.1080/19336950.2020.1740506

**Published:** 2020-03-18

**Authors:** Atousa Arbabian, Mircea Iftinca, Christophe Altier, Param Priya Singh, Hervé Isambert, Sylvie Coscoy

**Affiliations:** a Laboratoire Physico Chimie Curie, Institut Curie, CNRS UMR168, PSL Research University, Paris, France; b Sorbonne Université, Paris, France; c Department of Physiology and Pharmacology. Inflammation Research Network, Snyder Institute for Chronic Diseases and Alberta Children’s Hospital Research Institute (ACHRI), University of Calgary, Calgary, Canada; d Equipe Labellisée « Ligue contre le Cancer »

**Keywords:** Transient ﻿receptor ﻿potential (TRP) channels, invasion, evolution theory, gain of function, cancer

## Abstract

Transient receptor potential (TRP) channels form a family of polymodal cation channels gated by thermal, mechanical, or chemical stimuli, with many of them involved in the control of proliferation, apoptosis, or cell cycle. From an evolutionary point of view, TRP family is characterized by high conservation of duplicated genes originating from whole-genome duplication at the onset of vertebrates. The conservation of such “ohnolog” genes is theoretically linked to an increased probability of generating phenotypes deleterious for the organism upon gene mutation. We aimed to test experimentally the hypothesis that TRP mutations, in particular gain-of-function, could be involved in the generation of deleterious phenotypes involved in cancer, such as gain of invasiveness. Indeed, a number of TRP channels have been linked to cancer progression, and exhibit changes in expression levels in various types of cancers. However, TRP mutations in cancer have been poorly documented. We focused on 2 TRPV family members, TRPV4 and TRPV6, and studied the effect of putative gain-of-function mutations on invasiveness properties. TRPV channels have a C-terminal calmodulin-binding domain (CaMBD) that has important functions for regulating protein function, through different mechanisms depending on the channel (channel inactivation/potentiation, cytoskeleton regulation). We studied the effect of mutations mimicking constitutive phosphorylation in TRPV4 and TRPV6 CaMBDs: TRPV4 S823D, S824D and T813D, TRPV6 S691D, S692D and T702. We found that most of these mutants induced a strong gain of invasiveness of colon adenocarcinoma SW480 cells, both for TRPV4 and TRPV6. While increased invasion with TRPV6 S692D and T702D mutants was correlated to increased mutant channel activity, it was not the case for TRPV4 mutants, suggesting different mechanisms with the same global effect of gain in deleterious phenotype. This highlights the potential importance to search for TRP mutations involved in cancer.

## Introduction

TRP (transient receptor potential) channels are polymodal cation channels gated by thermal, mechanical, or chemical stimuli. In mammals, they form a subfamily of about 30 members, subdivided into 5 subfamilies, C (Canonical), V (Vanilloid), M (Melastatin), A (Ankyrin binding), PP (Polycystin) and ML (Mucolipin) [1,2]. TRP channels play an important role in various physiological mechanisms such as detection of pressure, temperature, taste, pain perception and vision, and mutations in these proteins are the cause of many genetic diseases, from skeletal dysplasias to neurodegenerative disorders, visual troubles, or cardiac genetic diseases (see Nilius et al. [3] for review). On the other hand, many TRP channels are involved in the control of proliferation, apoptosis, and cell cycle. TRP channels could be cancer biomarkers as changes in TRP expression have been associated with cancer progression. Structurally, TRP proteins are composed of six transmembrane domains, with TM4-5 forming the ion pore, and characteristic-conserved domains like TRP box (short sequence of amino acid near TM6), domains involved in protein interactions (*coiled-coil*, ankyrin, PDZ) and calmodulin-binding domains (for TRPVs and some TRPC).

From an evolutionary perspective, TRP families show a high conservation of duplicated genes originating from whole-genome duplication (so-called ohnologs), which are likely to be associated with the emergence of deleterious phenotypes upon mutations [4–7]. Indeed, following the two rounds of whole-genome duplication at the onset of vertebrate about 500 million years ago, each gene was present in up to four copies (ohnologs). During the following evolution period, vertebrate species had a natural tendency to get rid of supernumerary gene copies. However, a conservation of ohnologs is observed for specific gene families with 25–35% of ohnologs retained overall, but 60% for cancer-linked genes and 80% for genes with an auto-inhibitory activity [4–7]. Many of them are involved in “gain of function” phenomena, that are deleterious for the organism when mutated (cancer, genetic diseases). The rationale is that introducing random mutations to get rid of supernumerary copies generates deleterious phenotypes, leading to “purifying selection” (i.e. elimination of mutants), so that surviving organisms tend to retain non-mutated copies of “dangerous” ohnologs that are prone to dominant deleterious mutations. According to this scheme, we tested the hypothesis that mutating TRP channels resulted in deleterious phenotypes. We tested the gain of invasion, because of the general involvement of TRP members in cancer.

Indeed, a growing number of TRP channels have been reported to have a link with cancer [8–11] (TRPC1, 3, 4, 6, V1, 2, 6, M1, 2, 4, 7, 8, A1, PP1), with mechanisms that remain to be fully characterized but are related at least partly to their function in calcium signaling and its general role in transcription, cell cycle regulation, cytoskeleton dynamics, and cell contraction. Overexpression or underexpression of TRP channels was reported in many types of cancers, including breast, prostate, pancreas, colon, lung, and melanomas, and overexpressing TRP channels *in vitro* led to gain in proliferation [12–16], invasion [17–20] or changes in migration [21]. Strikingly, functional data published in the literature concern only changes of TRP expression level, and not somatic TRP mutations in cancers. (Note that, for some channels like TRPV1, V2, M1, M8, an additional regulation can be obtained through alternative splicing). Although some TRP mutations are found in systematic exome sequencing of tumors (COSMIC database) [22], it is not known whether they have any functional implication or significance in cancer development.

We focused here on the Vanilloid TRP subfamily, in which some members are linked to cancer (TRPV1, 2, 6, recently described for TRPV4) [23,24], and for which gain-of-function mutations are the cause of genetic diseases (TRPV4 [25]). TRPV6 is a selective calcium channel mainly expressed in small intestine, placenta, and pancreas, and participates in calcium reabsorption. It has long been known to be overexpressed in different types of cancers: breast, where it was proposed as a prognostic marker for aggressive breast cancers; prostate, with an oncogenic potential for TRPV6; colon, with a proposed protector role; thyroid, ovary, bladder, cervix, and uterus cancers [26–28]. TRPV4 is a polymodal nonselective cation channel involved in many physiological mechanisms, from control of osmotic pressure to vascular function, organ function, skeletal integrity, inflammation, and nociception. TRPV4 is mutated in several genetic diseases like skeletal dysplasia or neuromuscular diseases [25]. Links between TRPV4 and cancer have just begun to be documented. Recent reports show a decreased of TRPV4 expression in skin and bladder cancers [15,29], bladder and liver, and an overexpression in cervix, bladder, colon, lung, and uterus cancers [28], and TRPV4 is also involved in tumor cells angiogenesis [30]. TRPV4 was recently reported to be involved in breast cancer metastasis, through its action of softening the cell cortex by regulation of cytoskeleton/cortex proteins (actin/ERM/cadherin), by Ca^2+-^dependent activation of AKT and E-cadherin downregulation [20,31].

This study aimed to determine whether TRP gain-of-function mutations confer a gain in invasion. We focused on C-terminal calmodulin-binding domains (CaMBDs) that are present in C, V, and M subfamilies, and that are central in channel inactivation, potentiation, or cytoskeletal regulation, although with channel-dependent mechanisms. TRPV6 CaMBD contains a consensus sequence for PKC phosphorylation, with phosphorylation-decreasing calmodulin binding and slowing channel inactivation [32]. For TRPV4, C-terminal CaMBD (812–831) was proposed to be involved in calcium potentiation, *via* an auto-inhibitory mechanism coming from inhibitory interactions between N- and C-terminal domains, that would be released upon calcium entry [33,34]. Importantly, while TRPV6 does not directly interact with the cytoskeleton, TRPV4 has a dual binding to actin and tubulin via domain (798–831), superimposing with CaMBD domain. Phosphorylation of Ser824 (mediated by SGK kinase) has also been reported to be important for binding to actin and for and interaction with STIM1, regulating TRPV4 plasma channel density [35,36].

In this paper, we performed directed mutagenesis on TRPV4 and TRPV6 channels and showed that some gain-of-function mutations, in particular those mimicking a constitutive phosphorylation in the calmodulin-binding domain, increased cell invasiveness capacities. These results highlight the importance to study in more detail TRP mutations involved in cancer.

## Experimental procedures

### Reagents

N-((1 S)-1-{[4-((2 S)-2-{[(2,4-dichlorophenyl)sulfonyl]amino}-3-hydroxypropanoyl)-1-piperazinyl]carbonyl}-3-methyl-butyl)-1-benzothiophene-2-carboxamide GSK1016790A) was purchased from Sigma Aldrich Chemicals (St. Louis, MO). The drug was dissolved in DMSO at a stock solution of 100 mM and used for experiments at a concentration of 5 nM. The vehicle had no effect on the responses of the cells at the concentration used.

### Molecular biology

Plasmid hTRPV4-myc in pCDNA4-T0 was a kind gift of Nathalie Vergnolle and Corinne Rolland, TRPV6 in pIRES of Natalia Prevarskaya. Directed mutagenesis was performed using QuickChange Site-Directed Mutagenesis kit (Agilent Technologies). For electrophysiology, mutations were introduced in bicistronic pIRES2-DsRed2 (Clontech laboratories) vectors, allowing transfected cells to be selected for patch-clamp experiments.

### Cell culture and invasion assays

SW480 cells were purchased from ATCC and cultured in L15 + 10% FBS at 37°C, without CO_2_. Transient transfection was done by electroporation with a GenePulser II apparatus (measured transfection efficiency of 30% in our experimental conditions). Invasion assays were carried out in invasion chambers with Matrigel, 8 μm pores, of BD Biosciences. A total of 20,000 cells were seeded on invasion chambers, with 0% SVF on the top and 10% SVF at the bottom (after FACS selection of positive cells for TRPV4 experiments with bicistronic vectors). Cells were allowed to migrate 22 h, and invasive cells at the bottom were fixed with methanol and colored with cristal violet, while non-migrating cells on top were scratched away. Experiments were done in triplicates, with manual counting of all colored cells in each well. Error bars are *s.e.m.*

### Electrophysiological measurements

Tissue culture and transfection of human embryonic kidney cells (tsA-201) cells were carried out as previously described. Whole-cell patch-clamp experiments on HEK cells were performed 24 h after transfection. For recordings, cells were placed into a 2 ml bath containing (in mM): 140 NaCl, 1.5 CaCl2, 5 KCl, 2 MgCl2, 10 HEPES, 25 D-glucose, pH 7.4 adjusted with NaOH on the stage of an epi-fluorescence microscope (Olympus IX51, Olympus America Inc., Center Valley, Pennsylvania, USA). tsA-201 cells expressing the transfected TRPV4 wild type or any of the three mutants were identified via mCherry (red) protein fluorescence. Membrane currents were measured using conventional whole-cell patch clamp with pipettes pulled from borosilicate glass (Harvard Apparatus Ltd, UK) and polished to 2–4 MΩ resistance on a DMZ – Universal puller (Zeitz-Instruments GmbH, Germany). The pipette electrolyte contained (in mM): 120 CsCl_2_, 3 MgCl2,10 EGTA, 10 HEPES, 2 ATP, and 0.5 GTP, pH 7.2 adjusted with CsOH. Recordings were carried out using an Axopatch 200B amplifier and pClamp 10.4 software (Axon Instruments, Foster City, CA, USA). All solutions were prepared and all experiments were conducted at room temperature (22 ± 2°C). Data were filtered at 1 kHz (8-pole Bessel) and digitized at 10 kHz with a Digidata 1440 A A/D converter (Axon Instruments). Series resistance was 8.6 ± 0.9 MO before compensation (B85%), and average cell capacitance was 13.4 ± 2.3 pF. Only those cells that showed a stable voltage control throughout the recording were used for analysis.

## Results

### Conservation of ohnologs for TRP family members

**Figure 1.**
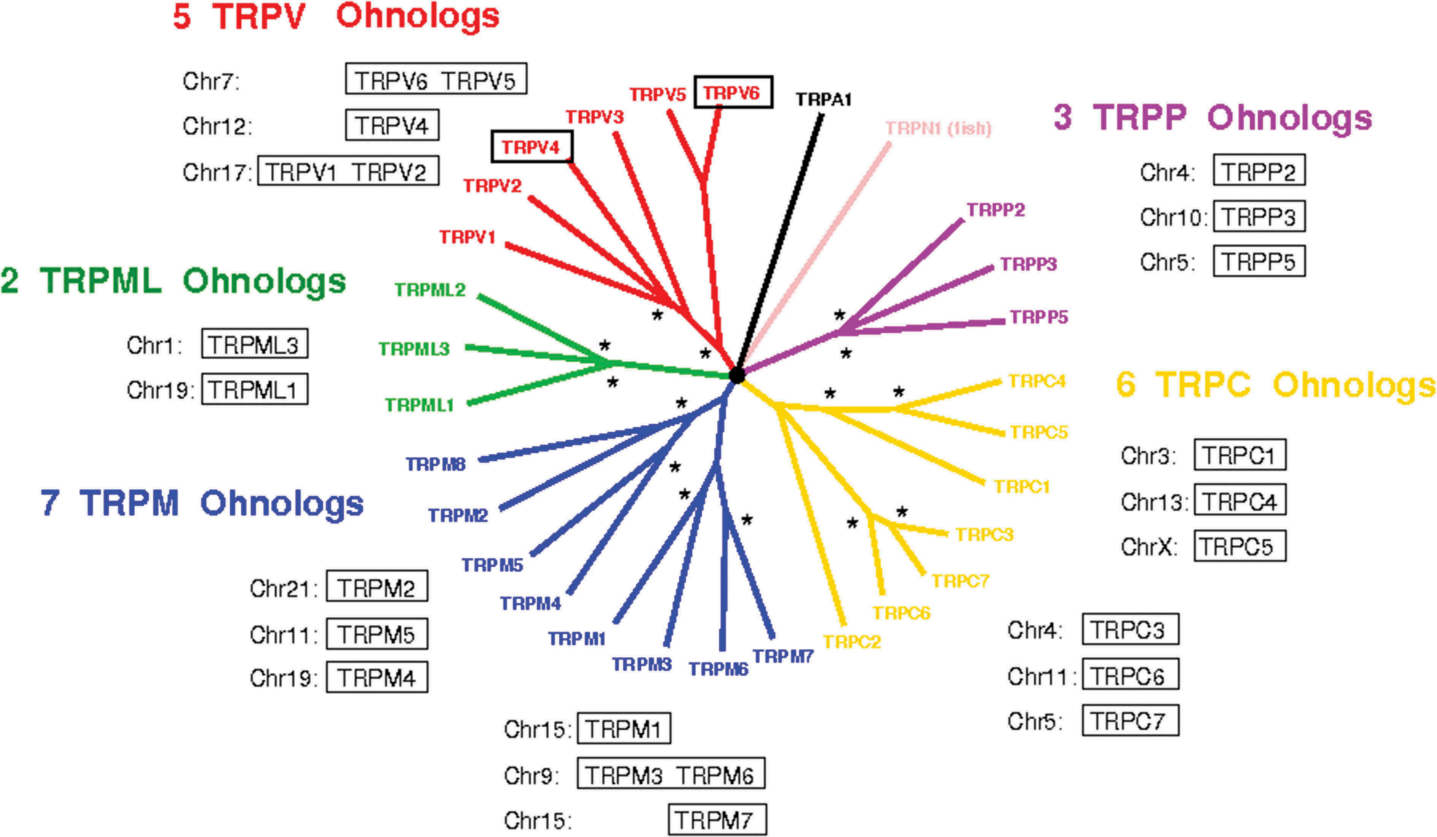
Ohnologs in TRP family. Phylogenetic tree was obtained from Nilius et al. [38], and asterisks correspond to the two whole-genome duplications on each branch (shown for ohnolog genes) [37].

Ohnologs of TRP channels have been determined using the method developed by Singh *et al.* [7,37] and the open-access online database at http://ohnologs.curie.fr/, Figure 1 (whole-genome duplications are marked with an asterisk on the phylogenetic tree). These results show that almost all TRP subfamilies exhibit a high degree of ohnolog conservation. The probability to be associated to deleterious phenomena upon gain-of-function mutations has been shown to increase with the number of ohnologs and becomes very important if three or four ohnologs are conserved. It is the case for TRPC, M, V, and PP subfamilies.

In this study, we focused on TRPV channels because (1) their CaMBD domain constituted a good target for generating gain-of-function mutations, and (2) a wide range of gain-of-function mutations were already reported for TRPV4 in the frame of genetic diseases. In TRPV subfamily, five ohnologs were found: TRPV6/TRPV5 (from a local duplication on chromosome 7), TRPV4 (on chromosome 12), and TRPV1/TRPV3 (from a local duplication on chromosome 17).

**Figure 2.**
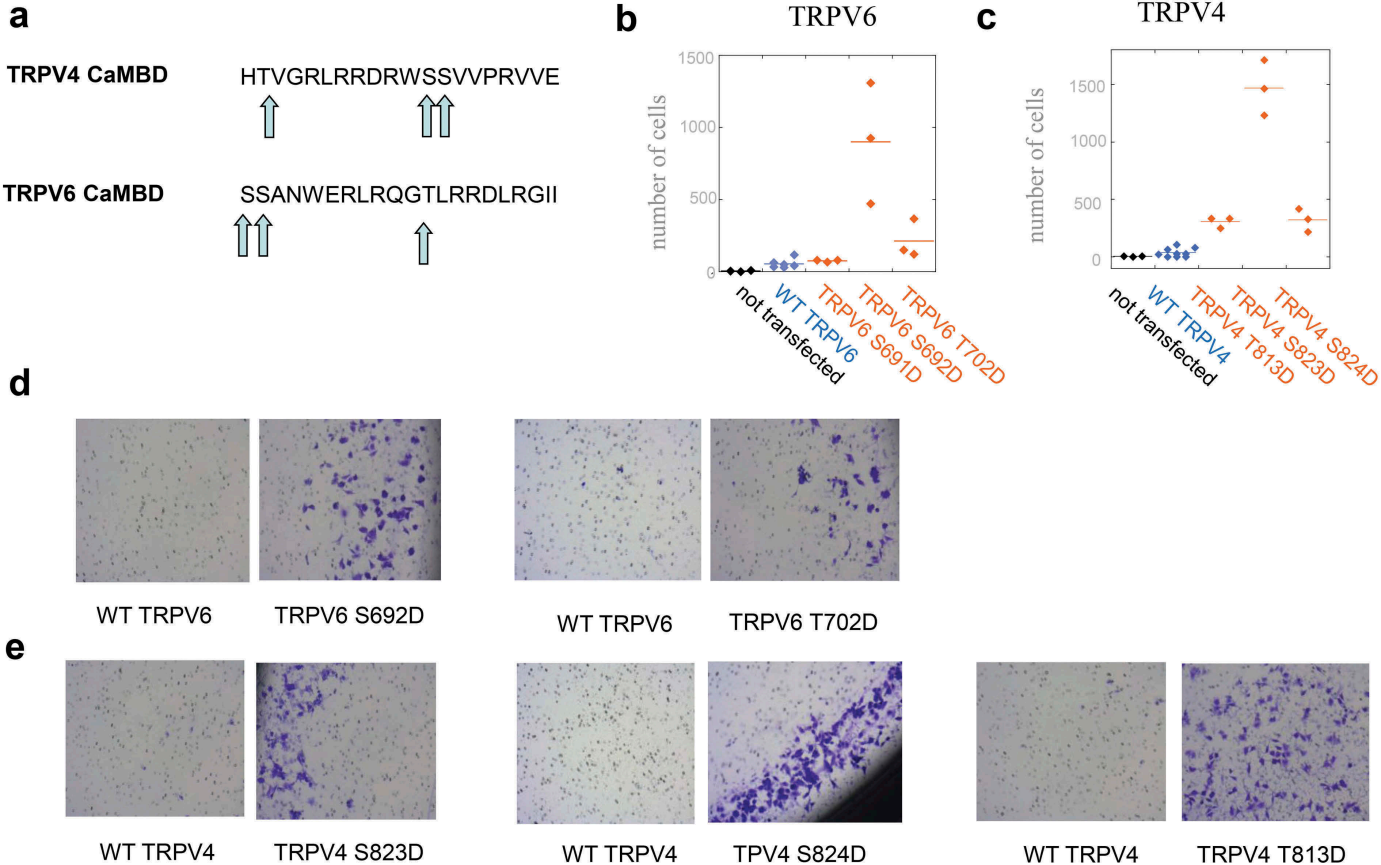
Mutations in TRPV4 and TRPV6 CaMBDs confer increased invasive properties to SW480 cells. (a) TRPV4 and TRPV6 CaMBD domains, with serine/threonine residues as potential phosphorylation sites (arrows). (b–c) Invasion assay on TRPV6 (b) and TRPV4 (c) CaMBD mutants mimicking constitutive phosphorylation. The number of cells passing through Matrigel after 22 h invasion (out of 20,000 seeded cells) is indicated. For TRPV4, bicistronic vectors allowing selection of transfected cells were used. A common reference (non-transfected cells) is indicated in (b) and (c) (black). (d–e) Typical images on cells harvested in invasion assays, on Corning BioCoat Matrigel invasion chambers with 8 μm pores, for TRPV6 (d) and TRPV4 (e). Images shown correspond to experiments with non-bicistronic vectors, both for TRPV6 (b) and TRPV4 (see SI).

A preliminary screen on gain in invasion upon known or putative gain-of-function mutations, most identified in genetic diseases, was performed for TRPV4 (not shown), highlighting the importance of mutations in CaMBD. We studied both TRPV4 (CaMBD involved in potentiation and cytoskeleton regulation) and TRPV6 (CaMBD involved in inactivation, no direct interaction with cytoskeleton). Phosphorylation on these CaMBD domains was reported to be important both for TRPV4 and TRPV6 activities; Figure 2(a) shows that each CaMBD contained three putative phosphorylation sites. We then performed systematic mutation in order to mimic their constitutive phosphorylation.

### Most gain-of-function TRPV4/6 mutations mimicking constitutive phosphorylation in CaMBD increase invasiveness of SW480 cells

TRPV6 contains a C-terminal calmodulin domain with two serines and one threonine (Figure 2(a)). Mutations TRPV6 S691D, S692D, and T702D, mimicking a constitutive phosphorylation in CaMBD, were performed. We quantified invasion by a classical assay on SW480 colon adenocarcinoma cells, very weakly invasive when non-transfected. While SW480 cells have an endogenous TRPV6 expression [39], TRPV6 expression already led to a small increase in invasiveness properties (Figure 2(b)). Two mutations, S692D and T702D, conferred a gain of invasion to TRPV6 when transfected in SW480 cells, compared to WT TRPV6 expression (Figure 2(b,d)). The number of invasive cells was 76 ± 5 (n = 3), 902 ± 240 (n = 3) and 213 ± 77 (n = 3) respectively, for S691D, S692D, and T702D, *vs* 55 ± 14 (n = 6) for WT TRPV6 control. These results suggest that, in SW480 *in vitro* invasion assays, mimicking constitutive phosphorylation of TRPV6 CaMBD confers invasiveness in two of three positions.

Like TRPV6 CaMBD, TRPV4 CaMBD contains two serines and one threonine (Figure 2(a)). Intestinal cells are not expected to express a significant level of endogenous TRPV4 [40], and expression of WT TRPV4 already conferred a slight increase in invasive capacity, as already reported [31]. TRPV4 S823D, S824D, and T813D conferred a strong gain in invasion (Figure 2(c,e)). After expression in bicistronic vector and selection of positive cells, the number of invasive cells was respectively 305 ± 29 (n = 3), 1467 ± 137 (n = 3) and 320 ± 57 (n = 3) for T813D, S823D, and S824D mutants, *vs* 36 ± 22 (n = 9) for the corresponding TRPV4 WT control (Figure 2(c)). Then, most CaMBD mutations mimicking constitutive phosphorylation in TRPV6 or TRPV4 channels strongly increased cell invasive properties of SW480 cells.

### Mutants correspond to gain-of-function in channel activity for TRPV6 and involve additional mechanisms for TRPV4

**Figure 3.**
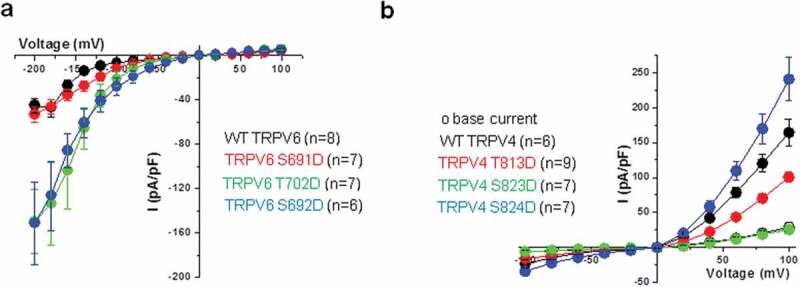
Electrophysiology of TRPV4/6 CaMBD mutants. Whole-cell patch-clamp experiments on HEK cells were performed 24 h after transfection with TRPV6 (a) or TRPV4 (b). Current–voltage relations obtained from the TRPV6 wild type and the three mutants (a) and for the TRPV4 wild type and mutant channels before (open symbol) and after GSK treatment (closed symbols). Each datapoint represents the mean ± SEM.

Channel properties of TRPV6 and TRPV4 CaMBD mutants were studied by whole-cell patch clamp, following expression in HEK cells devoid of endogenous TRPV6/4 (Figure 3). We found a gain of TRPV6 channel basal activity only for the two mutations generating a gain in invasion, S692D and T702D, both exhibiting a similar behavior (Figure 3(a)). The peak inward current of the TRPV6 WT channel (recorded at −200 mV) was smaller (−45.00 ± 6.59 pA/pF) compared to the T702D and S692D mutants which showed −149.4 ± 28.77 pA/pF (n = 7) and −151.35 ± 37.0 pA/pF (n = 6), respectively. This corresponds to a 332% (P = 0.001) and 336% (P = 0.001) increase in current for the T702D and S692D mutants, respectively (Figure 3(a)). As TRPV6 channels are strong inward rectifiers this suggested an increased calcium entry at resting membrane potentials that may constitute the source of calcium triggering the increased invasiveness of the cells. This indicates a possible link between gain-of-function mutations and dangerous phenotype, as predicted by the theory.

The situation was more complex for TRPV4. Base current was similar between the wild type and the three mutants (at: 28.87 ± 6.2 pA/pF, 14.94 ± 1.6 pA/pF, 15.11 ± 1.6 pA/pF, and 23.69 ± 4.7 pA/pF, respectively) (not shown). Exposure of TRPV4 wild-type expressing cells to the TRPV4 agonist GSK (5 nM) had a strong effect on the peak current (~600% increase, n = 6, P = 0.001) when compared to the baseline activity. However, only the TRPV4 S824D mutant showed an increase in the GSK-induced current over the wild type (~1000% increase compared to the base current, n = 7, P = 0.0003). The T813D mutant had less GSK-induced current compared to the wild type (although there was a ~ 650% increase over the base current, n = 9, P = 0.0001) and, finally, the GSK sensitivity of the S823D mutant was lost. ~170% (n = 7, P = 0.049) (Figure 3(b)). Of note, the GSK-induced effect was completely abolished after a 30-min wash (not shown). When compared to the TRPV4 wild-type peak current of 164.31 ± 19.1 pA/pF both TRPV4 S823D and S824D mutants showed statistically significant lower values at 25.5 ± 3.75 pA/pF (P = 0.00001) and 100.56 ± 7.43 pA/pF (P = 0.003), respectively (Figure 3(b)). The currents obtained with TRPV4 S824D mutant by depolarizing steps to +60, +80, and +100 mV were increased when compared to the wild-type (peak of 240.55 ± 31.16 pA/pF). These data reveal a robust and reversible GSK-mediated stimulation of TRPV4 wild-type, TRPV4 S824D and TRPV4 T813D currents but not S823D.

Therefore, none of the studied TRPV4 mutations led to a net increase in channel basal activity although upon stimulation S824D mutant showed, in our hands, an increase at more positive potentials over the wild-type channel similar to Shin et al. [35]. TRPV4 has been shown to participate in osmo- and mechanotransduction and this particular site has been shown, by Shin et al., to be implicated in the regulation of interaction with cytoskeleton proteins [35]. Furthermore, PKA-dependent phosphorylation of the channel at this site is involved in sensitization of the channel by hypotonic stimulations [41].

## Discussion

In this paper, we identified mutations conferring an invasive phenotype to the weakly invasive SW480 cell line. This study originated from evolution theory, suggesting indeed that TRP mutations, and not only overexpression or underexpression, may confer deleterious invasive phenotypes. Following a screening of different types of TRPV4 mutations, we focused on a centrally involved domain, CaMBD. It is interesting to note that this domain reported to have opposite roles (inactivation/calcium potentiation) for TRPV4 and TRPV6 channels in the literature [32,33], confers here identical invasion effects for both proteins.

The mechanisms of gain of function induced by CaMBD mutants are distinct between TRPV4 and TRPV6. TRPV6 mutants show increased basal activity (channel locked in open state). For this channel, the gain in invasion observed with our mutants is correlated with channel gain-of-function, very likely by loss of auto-inhibitory mechanisms previously described [27,42–44]. So mutations in TRPV6 could explain the invasiveness of the cells expressing mutant channel *via* increased basal activity.

For TRPV4, the gain in invasion is associated with increased channel activity only for S824D mutant: this consists in an increased activity in response to GSK (greater sensitivity or facilitated coupling between agonist binding and channel gating), and not an increased basal activity as for TRPV6 mutants. For this mutation, invasive effects could be explained by an endogenous agonist, such as arachidonic acid metabolite EET (or ROS compounds) [45] which have been involved in increasing the cancer cell migration. Alternatively, and for CaMBD mutations that did not lead to increased channel activity, mutations may be gain-of-function not because of direct modification of channel properties, but because of another property of CaMBD like cytoskeleton regulation [35] or control of channel density at the plasma membrane [36].

This would be in excellent agreement with recent published works on cytoskeleton/adhesion regulation by TRPV4 mediating breast cancer metastasis [20]. It was observed that WT TRPV4 overexpression led to increased breast cancer cell invasion, through regulation of cell stiffness, blebbing, and actin cortex [20]; the effects were mediated by Ca^2+^-dependent activation of Akt and E-cadherin down-regulation [31]. In light of these elements, the gain of invasion observed with our mutants may be due to increased actin binding and increased TRPV4 density at plasma membrane. Moreover, such mechanisms are highly suggested by the literature studying the role of Ser824 phosphorylation. First, Ser824 phosphorylation increases binding to actin [35]. Second, Ser824 phosphorylation also modulates interaction with STIM1 (stromal interaction molecule 1 precursor), regulating TRPV4 channel density [36]: TRPV4 S824D mutant cannot associate with STIM1, resulting in enhanced TRPV4 density in the plasma membrane. Given that overexpressed TRPV4 is sufficient to generate softening of cell cortex and an increase in invasion in breast cancer cells [20,31], this last mechanism may play an important role in the observed gain of invasion. While the precise mechanisms involved with the three CaMBD mutations studied go beyond the scope of this paper, we hypothetize that mimicking constitutive phosphorylation in TRPV4 CaMBD both has effects on channel density at plasma membrane and has direct effects of cytoskeleton regulation and signaling pathways (like Akt), leading to cortex softening and facilitated invasion [20].

Therefore, our data suggest that mutations in TRPV CaMBD domains lead to gain-of-function phenotypes through different pathways. They also establish that CaMBD phosphorylation sites appear as residues particularly sensitive to mutation in TRPV channels. However, other mutations outside the CaMBD domain or different mutations inside the CaMDB domains are also likely to be involved in deleterious phenotypes, as suggested by the variety of non-synonymous mutations associated with TRPV4 and TRPV6 in the COSMIC cancer database.
