# Supplementary material for: Mutations in calmodulin-binding domains of TRPV4/6 channels confer invasive properties to colon adenocarcinoma cells
Source: Channels (Austin). 2020 Mar 18;14(1):101–9. doi: 10.1080/19336950.2020.1740506 (PMC7153789; doi:10.1080/19336950.2020.1740506)
Supplement: Supplemental Material [file kchl-14-01-1740506-s001.docx]

**Supplemental Information:**

For TRPV4, invasion experiments were done both with bicistronic and non-bicistronic vectors (with or without selection of transfected cells, transfection efficiency 30-50%). Independent experiments with non-bicistronic vectors confirmed the strong gain in invasion for the three TRPV4 mutants, with corresponding quantifications 1210±315 (n=3), 630±22 (n=3) and 311±148 (n=6) for T813D, S823D and S824D mutants, *vs* 82±28 for WT TRPV4 (n=9)).
